# Multivariate statistics explaining groundwater chemistry, Asyut, Egypt

**DOI:** 10.1007/s10661-022-10338-8

**Published:** 2022-08-13

**Authors:** Ibrahim Said, Amr N. Abd-Elgawad, El-Montser M. Seleem, Salah A. M. Zeid, Salman A. Salman

**Affiliations:** 1 Geological Sciences Dept, National Research Centre, Dokki, Giza, Egypt; 2 El Minia Governorate Quarries Administration, Minya, Egypt; 3 Geology Department, Faculty of Science, Al Azhar University, Assiut Branch, Asyut, Egypt

**Keywords:** Groundwater, Anthropogenic influence, Multivariate statistics

## Abstract

Groundwater is an important source for domestic and irrigation purposes in Asyut area. Water quality varied widely due to complex geochemical processes and pollution sources. Understanding the processes controlling groundwater chemistry is necessary to overcome related problems. Multivariate statistics revealed that groundwater is affected by anthropogenic recharge (agricultural/organic pollution), mineralization, and redox processes. Contributions from natural vs. anthropogenic sources explain the variance in hydrochemical data. Shallow wells are relatively higher in bicarbonate content due to oxidation of organic pollutants. Shallow wells anomaly high with iron and organically polluted are most probably owing to pipe corrosion in residential areas. N fertilization impact on natural weathering has been demonstrated. Groundwater is getting more mineralized toward desert fringes due to lithological and hydrogeological characteristics under unconfined conditions. Evaporation factor enhances groundwater salinity under aridity. Fe and Mn contents are relatively higher as the redox potential is getting more reducing. The current study will help in building suitable management plan to protect the aquifer.

## Introduction

One of the major questions to address in groundwater resource management is to delineate the sources of solute in groundwater to inform the policy marker and resource manager. Therefore, the current study aims to identify the processes regulating groundwater chemistry to overcome related problems in Asyut region. Groundwater in arid regions is a wealth that must be preserved. Egypt suffers from water scarcity with a growing population and increasing demand. The per capita share/year does not exceed 600 m^3^; the total water shortage reached 42 BCM/y in 2018 (Mohamed, 2021). The current quota is expected to be depleted by 45% in 2050 (Mazzoni et al., 2018). Water scarcity is not only related to depleted quantity, but also to quality, as spoiled water is lost water. Therefore, defining the processes affecting water quality is of great importance. Groundwater quality is affected by agricultural and urban activities. Areas with pit latrines, sewage ponds, and/or cemeteries pose a risk to groundwater in terms of organic matter, bacteria, sulfate, chloride, and nitrates (Dissanayake & Chandrajith, 2009). Groundwater pollution due to the absence/deterioration of sewage systems and intensive fertilization has received attention in Egyptian literature (Redwan et al., 2020; Said et al., 2020; Ewida et al., 2021; Said & Salman, 2021; Salem et al., 2021).

Water chemistry varied widely due to complex geochemical processes and pollution sources in Asyut region. Overall understanding of geochemical processes regulating groundwater chemistry is necessary to overcome related problems. It is an initial step for the decision-makers through which they can make the most appropriate decision towards the problem by preventing, adapting, or avoiding it. Therefore, the research focused on identifying the hydrochemical processes controlling groundwater quality in Asyut area.

Multivariate statistics were applied to identify the relationship between groundwater variables and their origin. Principle component analysis (PCA) reduces the datasets to a few factors that are easy to interpret. PCA gets insight into the chief factors controlling water quality through the correlation between significant amounts of data. It is true that PCA does not provide a direct cause-and-effect relationship between environmental data (Yidana et al., 2012). However, it provides links between variables from which the main processes can be inferred (Said et al., 2021a); this requires good experience with the basic environmental issues. Aquifer mineralogy (i.e., water–rock interaction) controls the concentrations of the main components in the groundwater. Considering the role of mineralogy in groundwater chemistry, ions were modeled regarding their natural origin, using Gibbs (1970), end-member (Gaillardet et al., 1999) charts, and visual MINTEQ 3.1 (Gustafsson, 2011). The latter was applied to model dissolution/precipitation of minerals in groundwater. Piper (1944) plot helped to track the change in water facies and thus identify the dominant hydrochemical processes.

### Study area

**Fig. 1:**
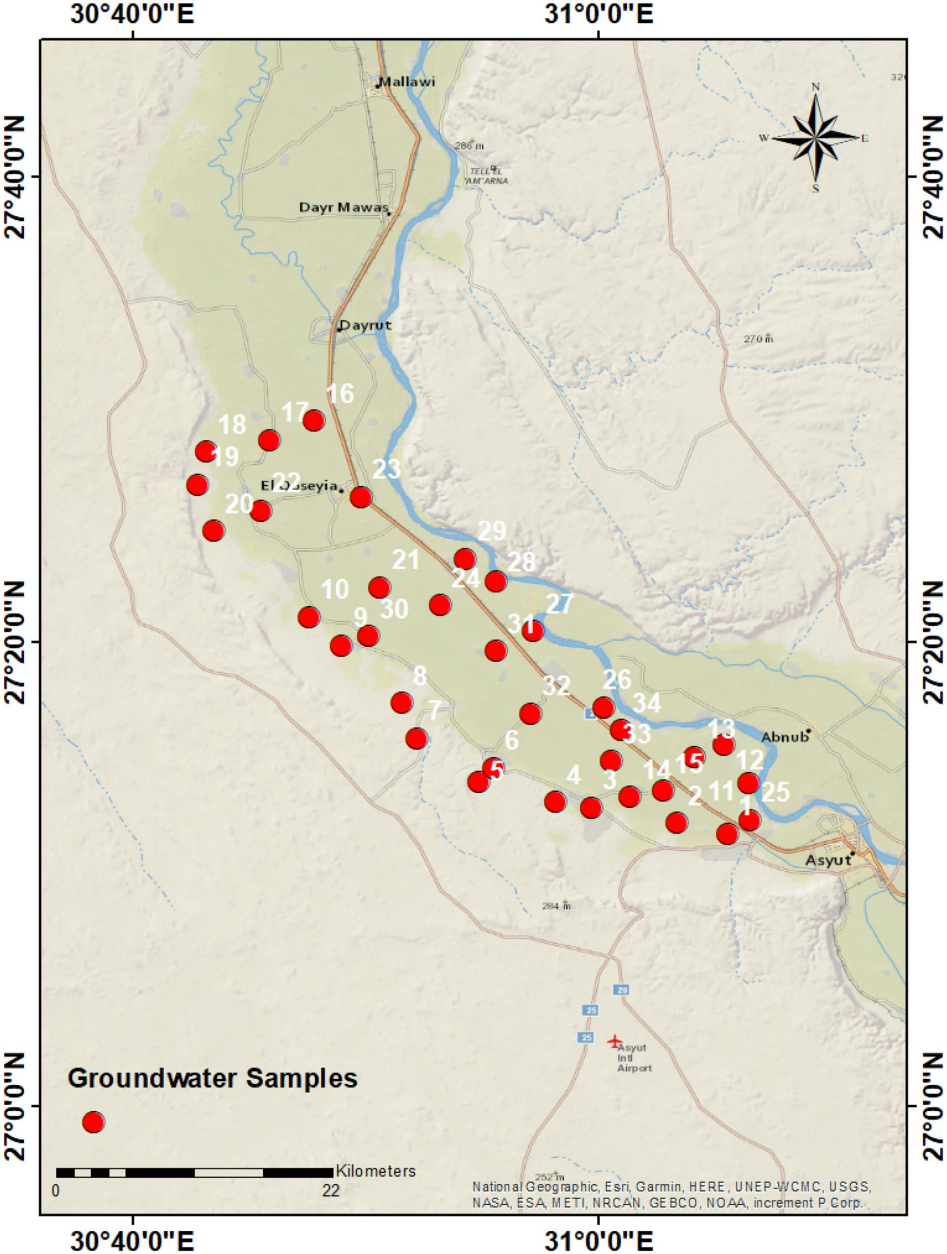
Sampled wells map at Asyut area

The study area lies between longitudes 26°50ʹ and 27°40 ʹN and latitudes 30°40ʹ and 31°32ʹ13.5ʺE, covering an area of about 700 km^2^ (Fig. 1) and is mainly occupied by agriculture and urban communities. The total population of Asyut Governorate is about 803,576 people, of whom 413,900 are located in the city, while the rest (389,676 people) reside in the villages (Seleem et al., 2021). Sewer networks are absent in most rural areas, and old urban networks are deteriorating. Residential complexes and scattered houses not connected to the public sewage network dispose of household waste either in private septic tanks (latrines) or directly into irrigation canals. As a result, groundwater quality is affected by urban and agricultural activities.

### Geology and hydrogeology

**Fig. 2:**
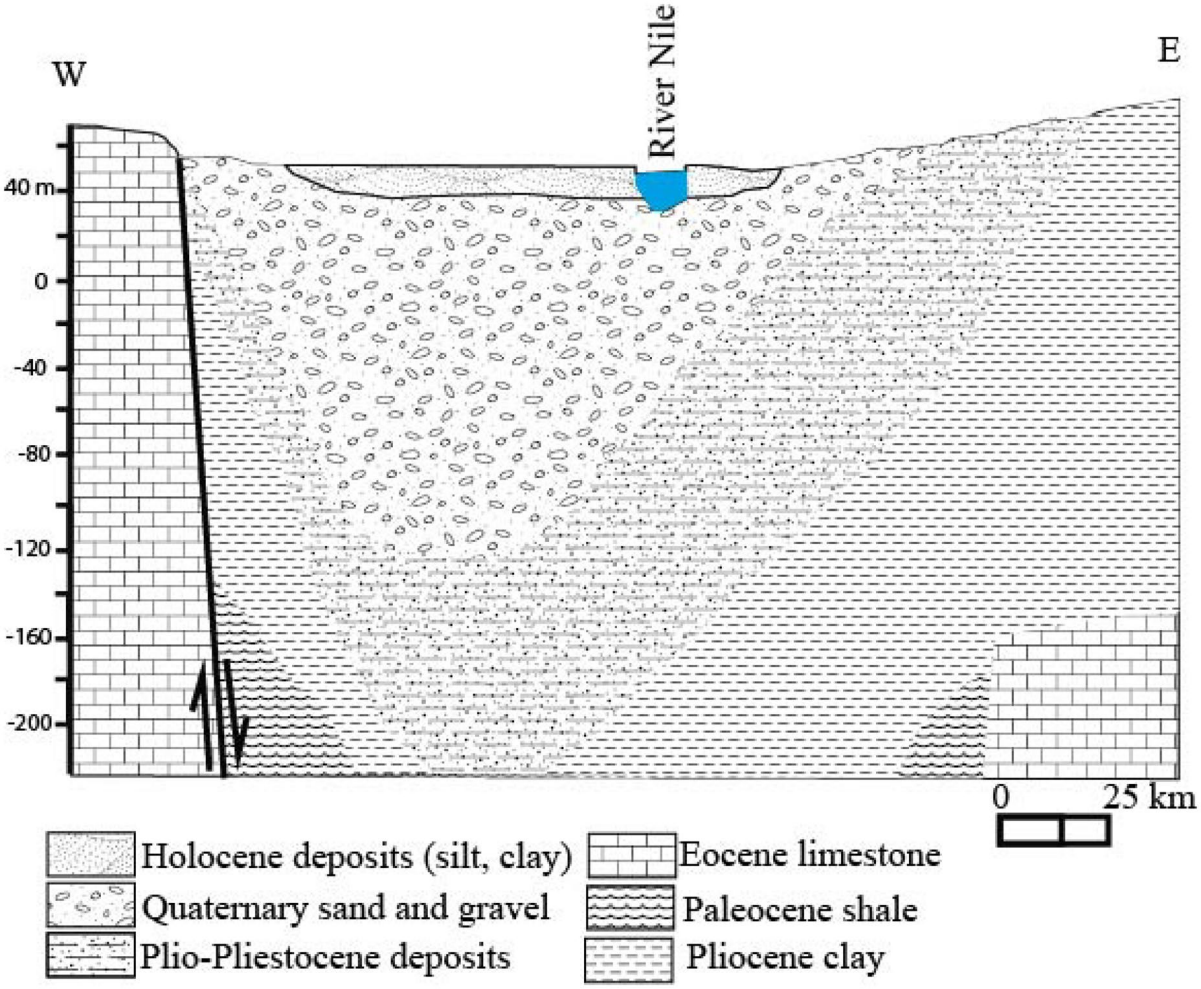
Hydrogeological setting at Asyut area (after RIGW, 1991)

Quaternary aquifer is the second water source after Nile for drinking and agricultural purposes. Geology and hydrogeology of the aquifer have been discussed in the literature (RIGW, 1991; Omer, 1996; Dawoud & Ewea, 2009; El-Rawy et al., 2021; Said & Salman, 2021; Said et al.,2021b), and summarized here as follows: Quaternary aquifer is meteoric in origin formed by rains. Figure 2 shows that the groundwater is provided from gravely sand Pleistocene deposits, sandwiched between Holocene clay-silt layer (aquitard) and Pliocene marine clay (aquiclude). Pleistocene water-bearing formations are distinguished into Qena and Kom Ombo formations under desert fringes and Ghawanim formation under floodplain. Holocene deposits (aquitard) differentiated into two sediment types: alluvial deposits (silt–clay layer) in the floodplain and Wadi deposits (clastic and carbonate sediments) in desert fringes. So, Holocene silt–clay layer absent in the desert forms an unconfined aquifer. The area is characterized by arid climate; temperature ranged from 18 to 43 °C, an annual rainfall of 3 mm with high evaporation rate (13.47 mm/month). Nile River and irrigation surplus are the main recharging aquifer sources. Leakage from septic tanks is an additional local source of recharge. Pumping wells are the main discharge source.

## Materials and methods

### Data collection and analysis

Thirty-four groundwater samples were taken from pumping wells in Asyut Governorate, Egypt (Fig. 1). Pre-rinsed polypropylene bottles were filled with the samples, sealed tightly. The temperature, pH, TDS, and electrical conductivity (EC) were determined in situ using a digital combined electrode (HANNA HI 991,300) which was calibrated prior to taking the readings. Redox potentiality (Eh) in mV was measured in situ using portable electrode (Hanna HI 98,120). In the laboratory, the samples were filtered using 45 µm and analyzed for chemical constituents. Sodium, potassium, sulfate, and nitrate were determined by spectrophotometer (HANNA HI 83,215). Total hardness (TH) as CaCO_3_, bicarbonate, and chloride were analyzed by volumetric methods. Iron and Mn were analyzed using the inductively coupled plasma-mass spectrometry (ICP-MS). Charge balances were calculated to verify the accuracy of chemical analyses; the error was less than 5% for all samples analyzed indicating excellent analysis accuracy overall. Chemical oxygen demand (COD) is the amount of oxygen consumed in the oxidation of organic components. COD values build upon a previous work by Abd-Elgawad et al. (2021) for the same wells.

### Statistical analysis and hydrochemical models

All statistical analyses were performed using SPSS 16.0 software. Descriptive statistics were conducted to understand the distribution of groundwater quality. PCA was performed to understand the main processes controlling groundwater quality, using varimax rotation (Kaiser, 1960), the most widely used method in the literature. Kaiser–Meyer–Olkin (KMO) and Bartlett’s test of sphericity were performed to assess the sample’s adequacy for PCA. KMO should be more than 0.5, and *P* value in Bartlett’s test should be less than 0.05. In this study, KMO = 0.7, above the minimum requirement of 0.5, with Bartlett’s test for sphericity of 0.00, below the significance level recommended by Tabachnick and Fidell (2007). This means that the data are suitable for PCA according to sampling adequacy tests (KMO and Bartlett’s sphericity tests). Furthermore, PCs that had an eigenvalue > 1 were kept, and the rest was removed. Variables with the highest significance (≥ 0.5) were selected to work on. This value (≥ 0.5) was taken as a baseline value to deal impartially with PCA results.

Given the contribution of aquifer mineralogy in groundwater chemistry, ions were molded regarding their natural origin using Gibbs and end-member plots. V. MINTEQ 3.1 has been applied to predict the dissolution/precipitation of minerals, in terms of their saturation index (SI). The mineral is in dissolution, equilibrium, and precipitation state with SI < 0, 0, and > 0, respectively. Piper (1944) diagram was created using RockWare Aq.QA software (V. 1.1, 2005). Based on the relative predominance of anions and cations, Piper diagram classifies groundwater into facies, which can be used to trace the water origin and hydrochemical processes such as ion exchange and water mixing.

## Result and discussion

### General hydrochemical characteristics

**Fig. 3:**
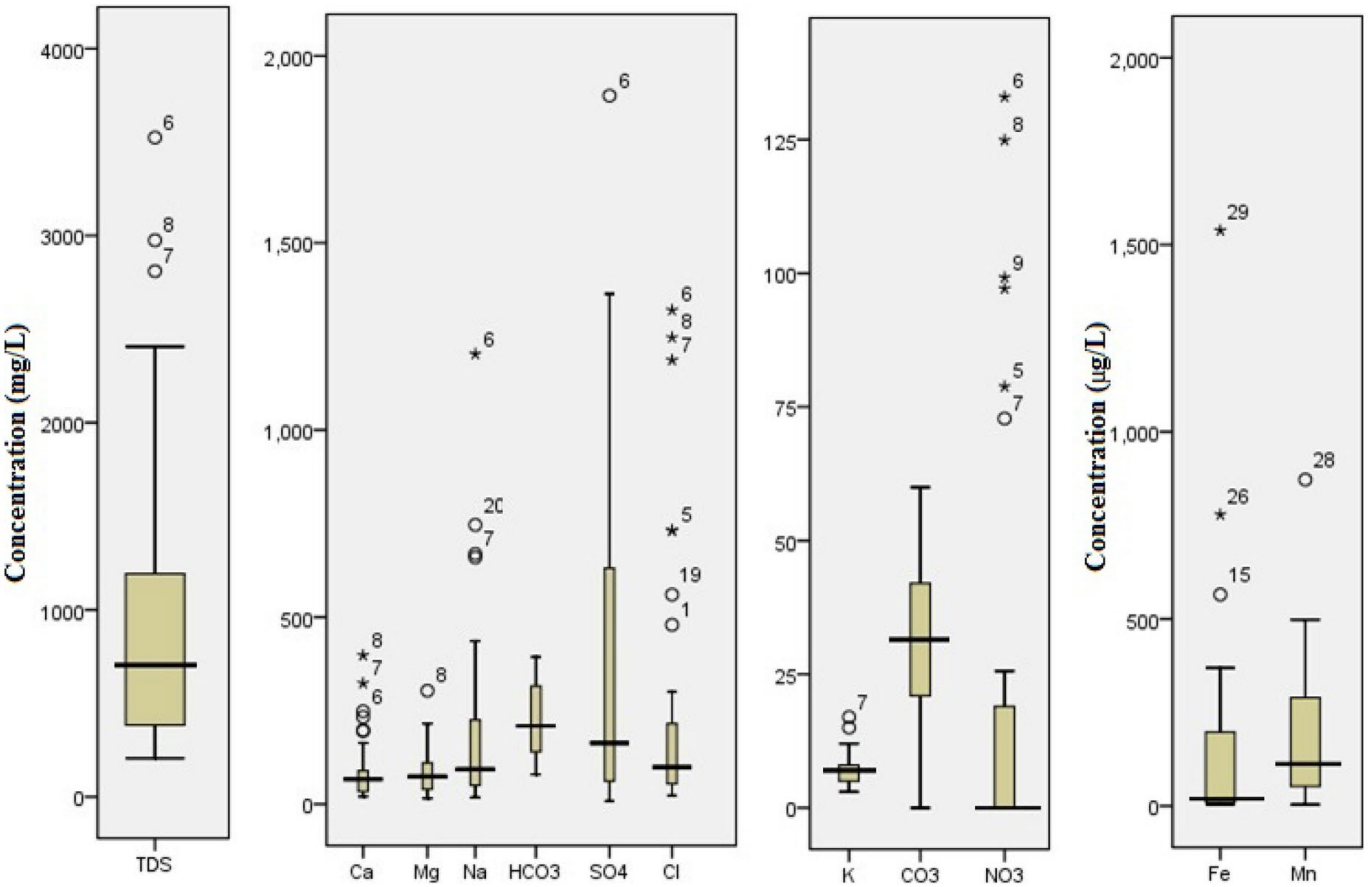
A box-whisker graph shows the variation of hydrochemical data in the aquifer system

Descriptive statistics of physio-chemical data are presented in Table 1 and Fig. 3, in comparison to WHO (2017) and FAO (1994) guidelines. Groundwater salinity ranged from 205.9 to 3524.3 averaging 991 mg/l. TDS variance (CV% = 86.64) suggests that groundwater salinity is controlled by mixed natural and anthropogenic processes. The 90 percentile data indicate that 10% of the dataset has TDS content above 2608 mg/l that exceeds recommended FAO (1994) value (2000 mg/l). Thirty-five percent of the water is undrinkable compared to WHO (2017). The samples displaying anomalous TDS values (outliers) are located close to the border of highly mineralized desert fringe under unconfined conditions, unlike most of the samples located in floodplains close to irrigation canals, under dilution effect. Accordingly, the water quality is affected by changes in lithology and hydrogeological characteristics. The mean pH value (7.3) is within WHO (2017) acceptable limit. The marked fluctuation of Fe and Mn content (CV% = 195.44 and 100.3, respectively) is controlled by the redox process; the matter goes in line with the great dispersion of Eh values (CV% of 308). The 90 percentile data indicate that only 10% of the dataset has Fe and Mn concentrations above 0.47 and 0.45 mg/l, more than WHO (2017) recommended value of 0.3 and 0.4 mg/l for iron and Mn, respectively. The anomalous Fe values (Fig. 3) in the residential communities refer to iron pipe corrosion. Organic pollution in shallow wells confirms such piping corrosion. The great variability of chloride is evidenced by the apparently scattered box-whisker graph (Fig. 3), in addition to the high interquartile range (IQR = 184 mg/l) representing 50% of the central samples. The stated vast in the chloride value suggested different processes controlling groundwater quality. The samples showing anomalous Cl^−^ values (outliers) are located close to the border of highly mineralized unconfined desert aquifer. Nitrate concentration varies from BDL to 133 mg/l. Nitrate concentrations exceed the desirable limit of WHO (2017) in 10% of the sampled wells. High nitrate content originated from agricultural activities and sewage effluents. All samples containing anomalously high nitrate levels (Fig. 3) are located in desert fringes, due to prevailing unconfined conditions and intensive fertilization. Sulfate content varies from 8 to 1893.8 mg/l with an average value of 380.6 exceeding WHO permissible limit. The great dispersion of sulfate (CV% = 152) clearly reflected in the scattered box-whisker graph (Fig. 3) implies anthropogenic influences, such as infiltrated domestic wastewater and agrochemicals. Average calcium concentration found within the normal range reported by FAO (1994) (0–400 mg/l) and higher than MPL in drinking water (75 mg/l). Na concentration falls within the usual range quoted by FAO (1994) (0–920 mg/l). The great variability of Na (CV% = 131.17) proposed that the hydrochemistry is controlled by intermixed factors. The mean concentration of Mg surpasses the usual range quoted by FAO (0–60.8 mg/l) and is still within the permissible limit for drinking water (100 mg/l). The interquartile range (IQR) of potassium points to that 50% of the samples have K content varying from 5 (lower quartile) to 8 mg/l (upper quartile) reflects limited fluctuation. Similarly, the low dispersion of bicarbonate suggests that natural processes mainly control groundwater chemistry whereas anthropogenic factors are limited.

**Table 1:** Descriptive statistics of groundwater data

**Variables**	**Min**	**Max**	**Mean**	**SD**	**Sk**	**Ku**	**5%**	**10%**	**L.q**	**Median**	**U.q**	**90%**	**95%**	**CV%**	**WHO** (2017)	FAO (1994)
T	20.6	29.8	24.3	2.2	0.3	0.0	20.68	21.25	22.62	24.55	25.72	27.65	28.98	9.17		
pH	6.9	7.9	7.3	0.3	0.4	− 0.7	6.93	6.98	7.07	7.3	7.49	7.7	7.84	3.59	6.5–8.5	6–8.5
TDS	205.9	3524.3	991.0	858.6	1.6	1.8	224.72	240	381.18	704.2	1306.65	2607.9	3111.95	86.64	1000	2000
EC	343.0	5856.5	1621.1	1397.9	1.6	1.9	363.17	393.35	624.32	1152.1	2140.82	4111.05	5154.12	86.23	1500	
Eh	− 118.0	572.0	52.5	161.8	1.3	2.1	− 115.75	− 111	− 77.75	4.5	136	251.5	457.25	308.11		
Ca	20.4	398.0	94.0	92.1	1.9	3.1	20.4	23.75	33.75	66.5	103	240.7	341.9	98.05	75	400.8
Mg	15.3	303.4	89.5	67.3	1.4	1.8	17.32	22.75	38.92	74.15	117.22	199.75	237.4	75.14	100	60.8
Na	18.0	1202.8	200.0	262.4	2.4	6.0	18.3	20.85	49.2	93.5	229.85	664.45	860.88	131.17	250	920
K	3.0	17.0	7.3	3.0	1.4	3.0	3	4	5	7	8	11	15.5	41.27	12	2
HCO_3_	79.3	393.5	226.7	91.2	0.2	− 1.4	97.6	114.4	139.55	208.95	317.08	354.9	370.62	40.23		610.1
SO_4_	8.0	1893.8	380.6	476.6	1.7	2.2	18.65	27.8	60.7	163.6	637.6	1262.55	1496.08	125.22	250	960.6
Cl	22.7	1320.2	259.3	364.4	2.0	3.1	23.68	33.45	52.07	99.1	236.45	959.2	1265.3	140.56	250	163.8
CO_3_	BDL	60.0	29.7	17.1	− 0.3	− 0.6	BDL	BDL	21	31.5	42	51	60	57.42		3
NO_3_	BDL	133.0	21.2	39.2	1.9	2.3	BDL	BDL	BDL	BDL	19.15	98.15	126.93	185.07	50	10
Fe	4.7	1537.7	155.0	303.0	3.4	13.3	8.66	10	10	18.96	198.67	467.3	968.85	195.44	300	5
Mn	3.8	872.6	189.5	190.1	1.7	3.7	5.84	8.95	51.41	112.24	295.02	446.95	591.58	100.31	400	200
Depth	3.0	170.0	58.3													

*Min*. minimum, *Max*. maximum, *SD* standard deviation, *Sk* skewness, *Ku* kurtosis, *L.q* lower quartile Q1, *U.q* upper quartile Q3, *10%, 90%* percentiles, *CV%* coefficient of variation, the number of samples (*n*) = 34, *BDL* below detection limit

### Hydrochemical pattern

**Fig. 4:**
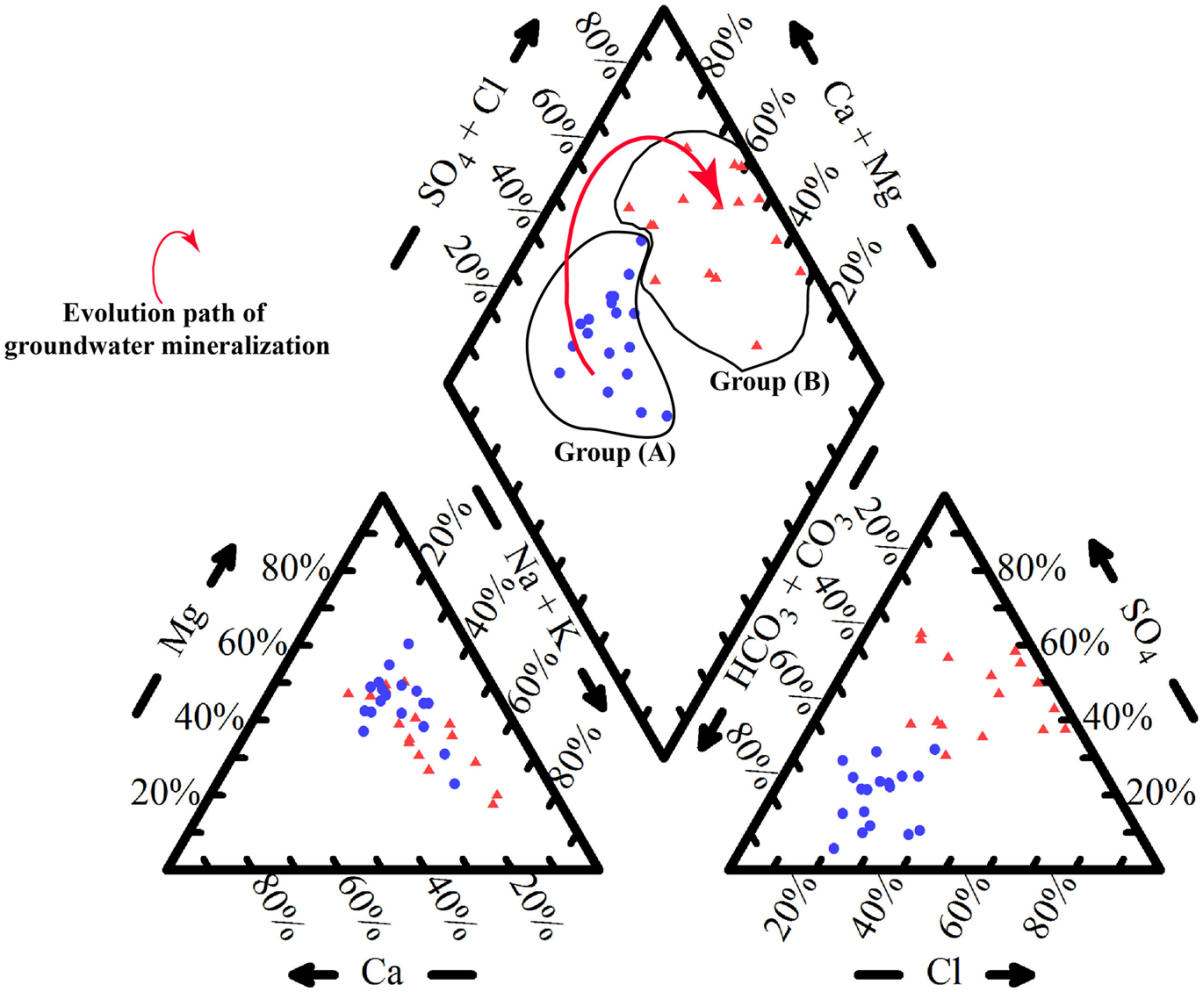
Groundwater recharge and mineralization processes

Groundwater types are Mg-HCO_3_ (43%), Mg-SO_4_ (18%), Na-SO_4_ (15%), Na-Cl (12%), Na-HCO_3_ (9%), and Mg-Cl (3%) facies. Piper plot (Fig. 4) showing that groundwater under floodplain (group A) is mainly of bicarbonate facies affected by surface recharge from river Nile, irrigation surplus, and sewage leakage, changed toward desert fringes (group B) into chloride and sulfate facies with higher salinity, i.e., water types changed laterally from low mineralized floodplain aquifer to highly mineralized unconfined desert aquifer due to different lithology, hydrological features, and land use (Said et al., 2020; Said & Salman, 2021). The matter confirms effects of natural and anthropogenic factors on the groundwater quality.

### Natural processes controlling water composition

**Fig. 5:**
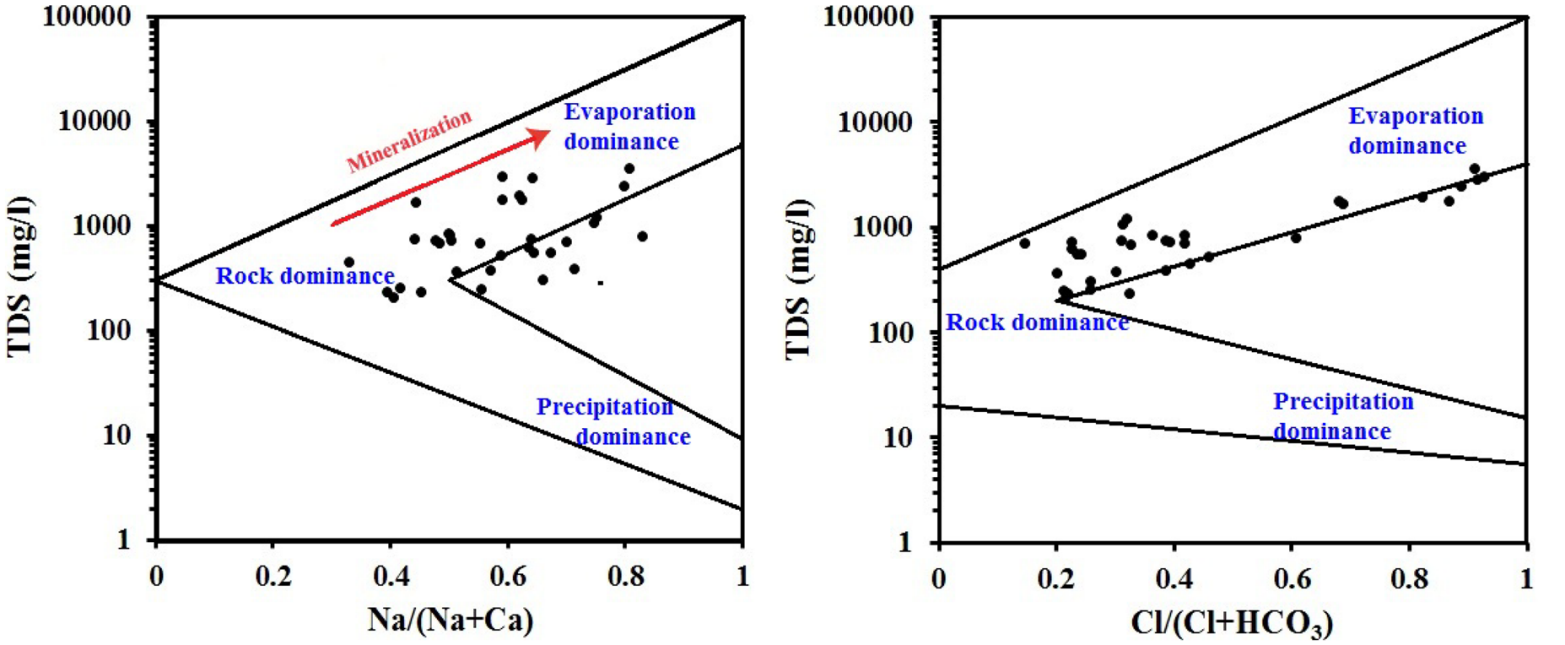
Natural weathering input in groundwater composition

**Fig. 6:**
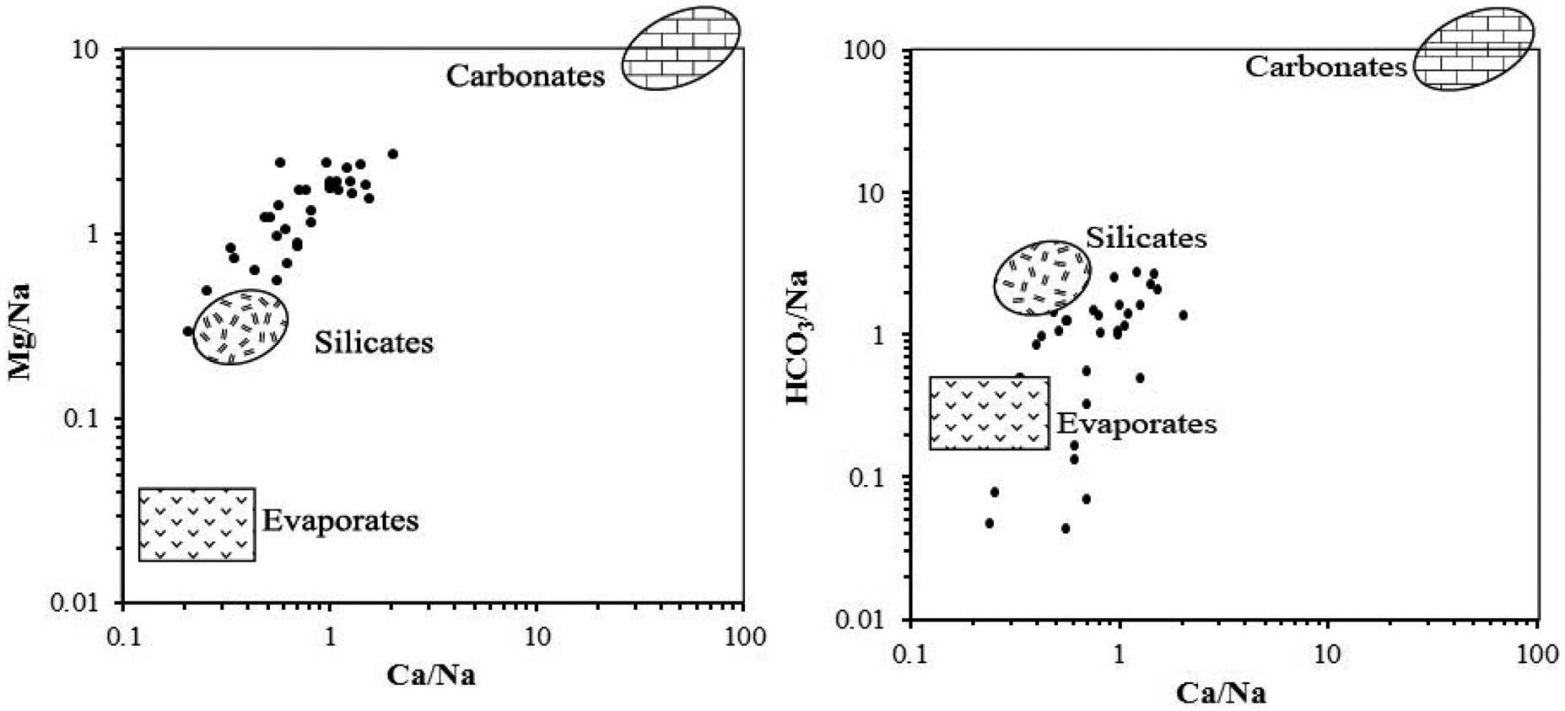
Relative contribution of silicate and evaporates weathering in groundwater

Considering the role of aquifer mineralogy in groundwater chemistry, ions were modeled regarding their natural origin using the hydrochemical graphs (Figs. 5 and 6). Gibbs diagram (Fig. 5) indicated that groundwater composition is controlled by the aquifer mineralogy, evaporation process, and anthropogenic impact. Samples fall under rock dominance area implying that the rock-water interaction is the most dominant process controlling groundwater chemistry. The ratio of Na / (Na + Ca) > 0.5 on Gibbs diagram indicates ion exchange process (Li et al., 2020; Said et al., 2021b). Another important factor impacting groundwater chemistry is the evaporation process as a result of aridity. Evaporation process increases water salinity under arid and unconfined conditions through a loss of water increasing salt concentration. Samples falling outside the plot preview point to anthropogenic influences (Ramachandran et al., 2019).

Silicate and evaporate weathering are responsible for the evolution of groundwater composition (Fig. 6). Hydrogeochemical data point to the weathering of aluminosilicate minerals as a major lithogenic contributor to Na, K, Ca, and Mg. Regional geology (Omer, 1996) favors silicate weathering as a potential source of these cations, where plagioclase, mica, amphiboles, pyroxene, etc. are predominant minerals in the aquifer sediments. Multivariate statistics explained the role of nitrogen- and sulfur-bearing materials (agrochemicals) in silicate weathering. The general reaction of silicate weathering with nitric acid can be written as:

Mg.Na.Ca.K─silicates + HNO_3_ = H_4_SiO_4_ + NO_3_ + Mg‏ + Na + Ca + K + solid product.

Similarly with sulfuric acid as:

Mg.Na.Ca.K─silicates + H_2_SO_4_ = H_4_SiO_4_ + SO_4_ + Mg + Na + Ca + K + solid product.

Dissolution of evaporite minerals was confirmed using V. MINTEQ 3.1, where halite, gypsum, and anhydrite are undersaturated in all sampled water with saturation indices values averaging − 6.3, − 1.7, and − 1.8, respectively.

### Multivariate statistics

**Fig. 7:**
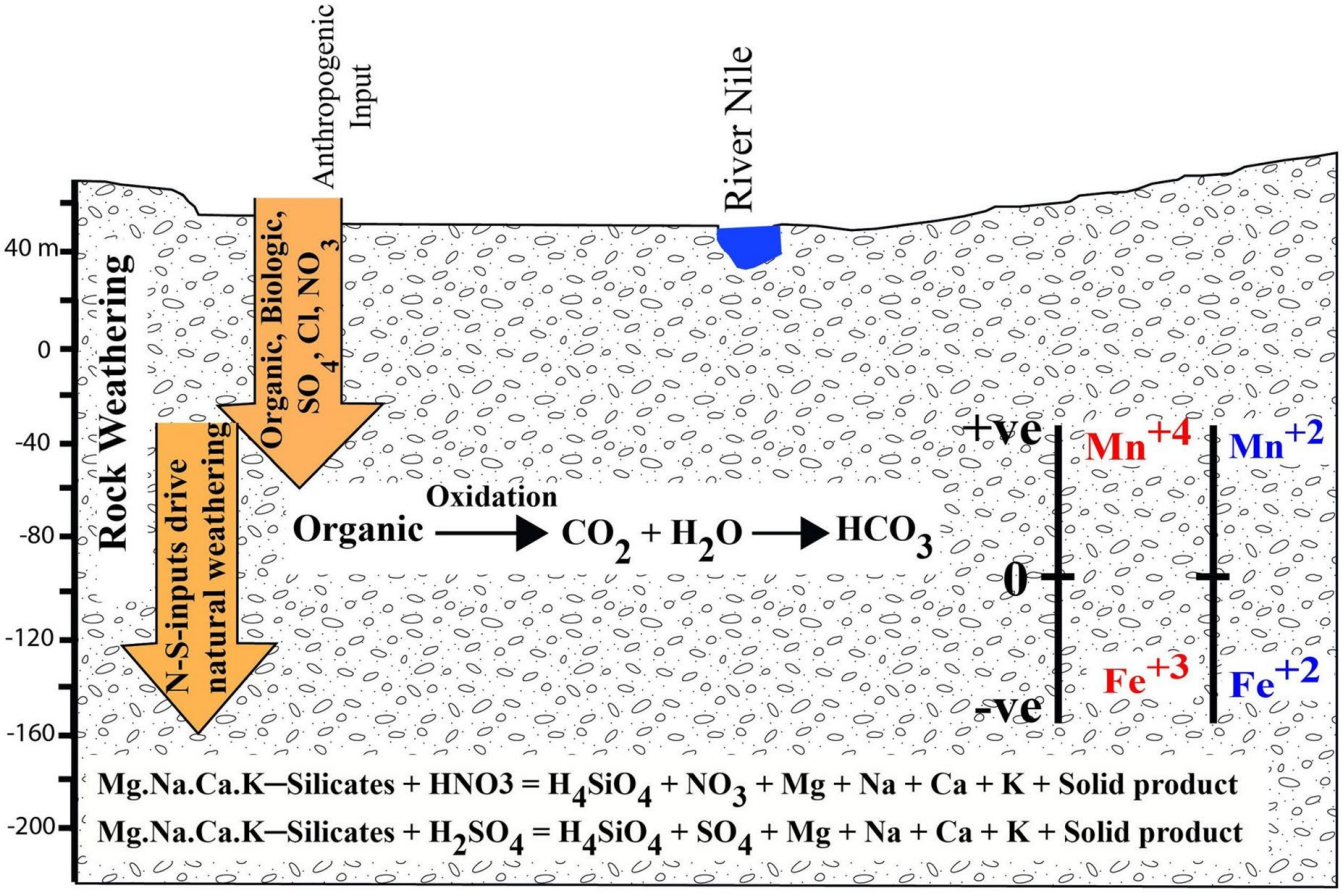
Schematic mechanism of the processes controlling groundwater chemistry

To understand groundwater chemistry, PCA was performed using varimax rotation method. Three rotated components representing 74% of the dataset explain the processes controlling groundwater chemistry (Table 2; Fig. 7). The first rotated factor (PC1) explains 47% of the total variance, showing a strong association between TDS and major ions in an inverse relationship with pH. Accordingly, PC1 can be considered as a mixed factor explaining the natural weathering due to acidification under N fertilizer application. That is, PC1 has a mixed origin as a result of the interaction between natural and anthropogenic factors. The high variance of ions loaded in PC1 and anthropogenic marker of NO_3_^−^ denote anthropogenic influences. The presence of NO_3−_ supports anthropogenic contribution from agricultural activities (N-fertilizers). Piper diagram (Fig. 4) indicates that the hydrochemistry of groundwater is influenced by recharging from irrigation return flow and sewage effluents. On the other hand, these components are present in normal concentrations, signifying their natural origin. Gibbs and end-member diagrams (Figs. 5 and 6) suggest that the chemistry of major ions is regulated by silicate and evaporite weathering. Accordingly, the concentrations in groundwater are coming from mixed sources of solute loads (natural and anthropogenic sources).

**Table 2:** Rotated component matrix of groundwater data

Variable	Principal component loadings		
	**PC1**	**PC2**	**PC3**
TDS	.966	.186	.092
Ca	.920	.111	.201
Mg	.948	.088	.038
Na	.906	.299	.139
K	.897	.028	− .080
HCO_3_	− .061	− .434	− .774
SO_4_	.863	.300	.113
Cl	.919	.185	.285
NO_3_	.785	.365	.182
COD	− .053	.235	− .517
Fe	− .092	− .576	− .055
Mn	− .112	− .787	.004
Eh	.251	.658	− .043
pH	− .684	.235	.435
Depth	.193	.018	.802
Eigenvalues	7.687	2.036	1.375
Variance%	47.378	13.903	12.714
Cumulative%	47.378	61.281	73.995

Extraction method: principal component analysis. Rotation method: varimax with Kaiser–Meyer–Olkin normalization (KMO = 0.7). Rotation converged in 5 iterations

PC2 explains 13.9% of the variance and exhibits Fe and Mn in negative loading with Eh. Fe and Mn concentrations are relatively higher as the redox potential is getting more reducing. This conclusion indicates that Fe and Mn contents are controlled by the redox process and not by compositional/textural features as discussed by Omer (2003), who found that Fe and Mn content increases upwards towards the finer sediments and Ghawanim formation rich in heavy minerals. PC2 can be denoted as a redox factor. PC3 is responsible for 12.7% of the variance, showing COD and HCO_3_^−^ negatively correlated with depth, implies organic pollution is more effective in shallow wells and declined gradually downward. Oxidation of these organic pollutants is the origin of high bicarbonate levels in shallow wells. Oxygen is consumed by organic pollutants producing carbon dioxide and H_2_O; their combination gives HCO_3_^−^. Sewage pipe corrosion inferred above confirms this organic pollution.

## Conclusion

Groundwater chemistry determines its suitability for various purposes. Hence, defining the hydrochemical processes regulating groundwater chemistry is necessary to overcome related problems. In this regard, the hydrochemical parameters were analyzed to get insight into the natural and anthropogenic processes affecting groundwater chemistry in Asyut region. Groundwater is influenced by anthropogenic contributions viz agricultural activities and organic pollution. N fertilization enhances the natural weathering. Iron and Mn contents are mainly controlled by the redox process. The water types vary spatially from HCO_3_ (in floodplain aquifer) to SO_4_ and Cl (in the desert aquifer) due to different lithology, hydrological features, and land use. The current study not only gets insight into water quality assessment but also addressed the sources of pollution to facilitate environmental management for decision-makers. As the utilization of geochemical processes in pollution management is the specialty of environmental geochemistry. Hence, it is recommended to periodically maintain the sewage system in Asyut Governorate to avoid organic pollution and increased iron concentrations in groundwater as a result of sewage pipe corrosion. It is also recommended to use eco-friendly fertilizers.

## Data Availability

The data that support the findings of this study are available from the corresponding author upon request.
